# Hospital Sunday Sermons

**Published:** 1904-06-18

**Authors:** 


### HOSPITAL SUNDAY SERMONS.

It is impossible to give quotations from all or even from many of the sermons in which the church-going public were besought to contribute to the maintenance of our medical charities on the 12th, for the cause of our hospitals was pleaded in almost every pulpit in the metropolis and suburbs, but we have culled a few words from some of the most prominent of the preachers. The Lord Mayor, accompanied by Sheriff Sir Alfred Reynolds and several aldermen, was present at Divine Service at the City Temple in the morning, and at St. Paul's in the afternoon, when the Bishop of Stepney preached the sermon.

The Bishop quoted the dictum of an Eastern visitor to the Coronation that London was "part beautiful, part awful," and gave as an example of the awful side of the great city the suffering which, but for the existence of our hospitals, must remain unrelieved. He reminded his hearers how modern methods and conditions of labour tended to separate class from class, and keep the rich ignorant of the sufferings of the poor. The existence of hospitals tended to bridge the gulf. He protested against the idea of making the hospitals of London dependent upon public money, because this would snap the last tie that bound the wealth of London to the spirit of stewardship. He did not doubt that under any conditions doctors and nurses would do their work as nobly as they do now, but what was at stake was the moral self-respect of London. A kindred string was touched at the evening service, when the Bishop-Designate of Brisbane declared that "the presence of the sick was a moral asset to the community."

At Westminster Abbey Canon Welbore MacCarthy, who preached the morning sermon, expressed the opinion that there must be many who could give substantially to this great annual effort to supply the multitude of the sick poor of London with medical and surgical aid; while in the afternoon Bishop Welldon reminded his hearers that although it was almost certain that nature contained a remedy for every disease, the task of finding out these remedies was always arduous and costly, and commended the cause of the hospitals on the ground of their services to medical science as well as to their patients. In the evening Archdeacon Wilberforce said that the Christian revelation affirmed that for purposes of amelioration every human being was a healer, but pointed out that in many cases the function could only be carried out by delegation to the medical profession and the hospitals. To imagine London without its hospitals would be to imagine a wilderness of hopeless pain.

At St. Saviour's, Southwark, the Bishop of Richmond gave it as his opinion that the hospital movement was as hopeful and helpful to the spirit of brotherhood as any movement of this generation. A similar idea was in the declaration made by the Rev. R. J. C. Campbell, at the City Temple, that there was no sign of unity so hopeful to-day as the unity of the Churches in this appeal for the hospitals. Mr. Campbell also said that "no one rejoiced more than his Majesty's subjects that the Royal Family was first and foremost in such agencies as those instituted for the relief of God's poor."

At the Great Synagogue the Chief Rabbi, in the course of a very successful appeal to his congregation, stated that there were grave arguments against the taking over of hospitals by the State. He had been assured that to do so would entail the levying of a special rate of about two shillings in the pound. Moreover, our hospitals would no longer be sermons in stones, preaching the golden lesson of spontaneous benevolence, and our Royal House would no longer be able to be the most illustrious exemplars of devoted service in the cause of the sick poor, realising, as they did at present, the fulfilment of the Divine promise, "Kings shall be thy nursing fathers and queens thy nursing mothers."

